# P-527. Impact of Obesity and adipokine levels in Young Children with Respiratory Syncytial Virus (RSV) Infection

**DOI:** 10.1093/ofid/ofaf695.742

**Published:** 2026-01-11

**Authors:** Helena Brenes-Chacon, Kendall T Whitt, Cristina Tomatis Souverbielle, Marie Wehenkel, Cristina Garcia-Maurino, Stacey Schultz-Cherry, Octavio Ramilo, Asuncion Mejias

**Affiliations:** St. Jude Children's Research Hospital, Germantown, TN; St. Jude Children's Research Hospital, Germantown, TN; Nationwide Children’s Hospital, Worthington, Ohio; St. Jude Children's Research Hospital, Germantown, TN; Universitat de Barcelona, Barcelona, Catalonia, Spain; St Jude Children's Research Hospital, Memphis, Tennessee; St. Jude Children's Research Hospital, Germantown, TN; St Jude Children's Research Hospital, Memphis, Tennessee

## Abstract

**Background:**

Obesity has been linked to worse clinical outcomes in children infected with influenza and SARS-CoV-2. The limited number of studies examining the relationship between weight and disease severity in young children with RSV infection have primarily focused on the role of undernutrition.

**Figure 1.**
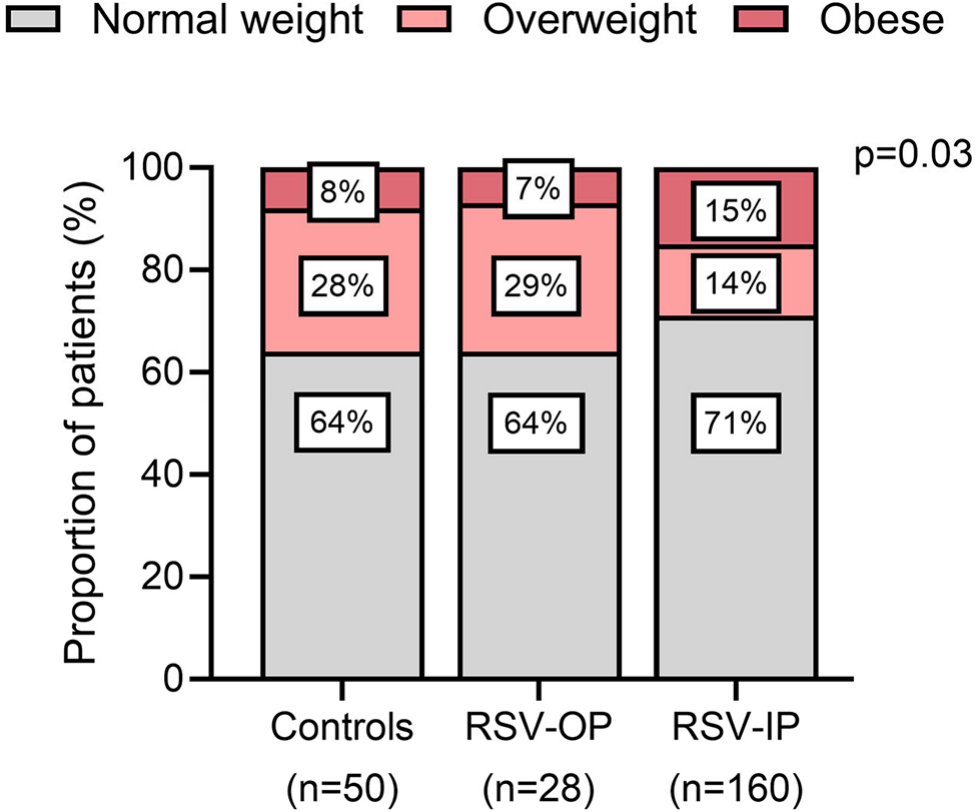
Nutritional status among infants with mild (outpatients) and severe (inpatients) RSV infection and healthy controls X axis includes the three clinical groups, healthy controls, children with mild RSV infection– outpatients; OP– and children with severe RSV infection (inpatients- IP0) that were stratified according to their nutritional status (gray: normal weight, light pink: overweight, dark pink: obese). Y axis represents the proportion of patients included in each category.

**Table 1.**
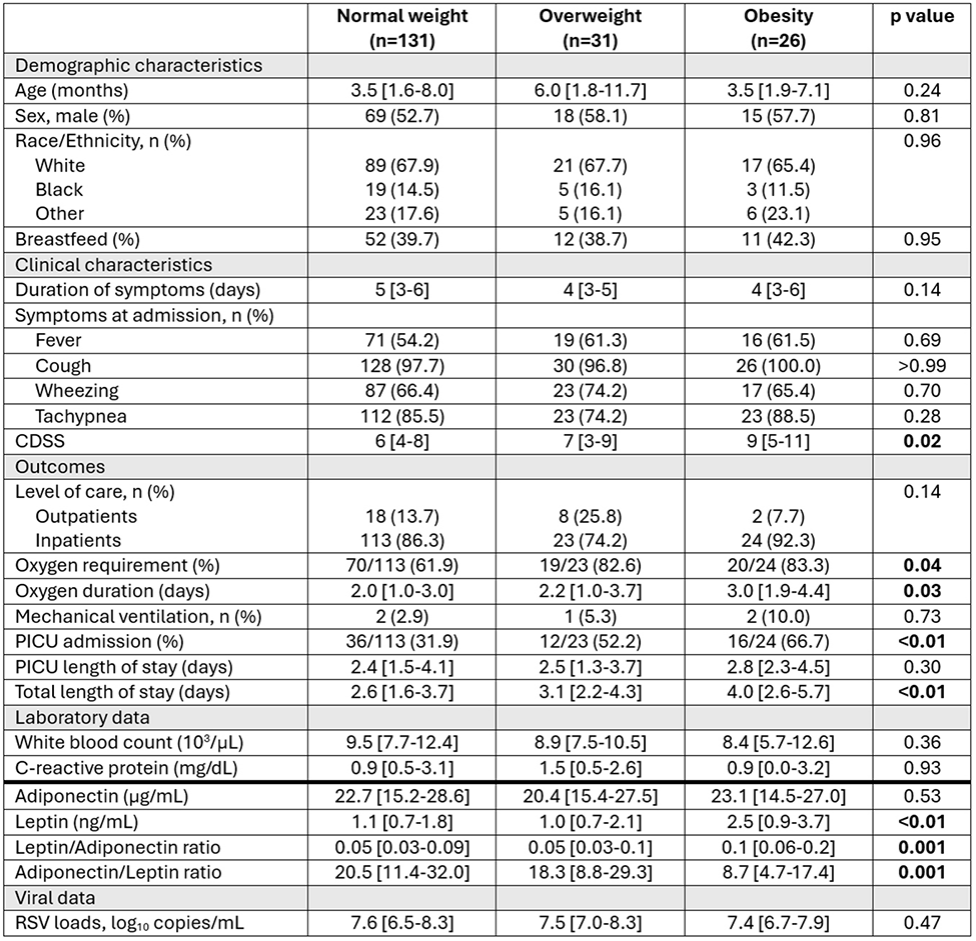
Demographic, clinical characteristics, and outcomes of children with RSV infection according to their nutritional status. CDSS: clinical disease severity score. Data expressed as median [25-75% IQR] for continuous variables and as numbers (%) for categorical data. Analyses by Fisher or Chi-square, and Kruskal-Wallis test with Dunn’s multiple comparisons test.

**Methods:**

Prospective observational study in otherwise healthy children < 2 years hospitalized or managed as outpatients with RSV infection at a large US Pediatric Hospital between 2015-21. Healthy control infants were enrolled in parallel. Nutritional status was assessed using the WHO Z-scores for weight and length according to age. Normal weight (eutrophic) was defined as −0.9 to 0.9 SD, overweight as 1.0 to ≤2.0 SD, and obesity as >2 SD. Overnutrition included the combination of overweight and obesity. Serum leptin and adiponectin concentrations were measured by ELISA, as regulatory molecules related to metabolism and weight, and correlated with clinical outcomes.

**Results:**

We enrolled 188 children with RSV infection (median [IQR] age: 4.2 [2.0-8.6] months), and 50 healthy controls (7.8 [5.3-13.3] months). Obesity was found in 7% of RSV-outpatient infants, 15% of RSV-inpatients and 8% of controls; p=0.03; Fig 1. Within infants with RSV infection, those with overnutrition required supplemental oxygen more frequently (83% vs. 62%; p< 0.01) and had higher rates of PICU admission (60% vs. 32%; p=0.001) compared to those with normal weight. Moreover RSV-infected infants with obesity had higher clinical disease severity scores, greater need for PICU admission, longer hospitalizations, higher leptin concentrations, and greater leptin/adiponectin ratios compared with eutrophic and overweight RSV-infected children (p< 0.01); Table 1. There were no differences in C-reactive protein concentrations and RSV loads between normal vs overweighted children.

**Conclusion:**

Excess nutrition in infants and young children assessed by Z-scores, showed differences in leptin/adiponectin concentrations, and was associated with greater RSV disease severity in a high-resourced country. This study underscores the importance of monitoring not only undernutrition, but overweight and obesity in young children with RSV infection.

**Disclosures:**

Cristina Tomatis Souverbielle, MD, Merck inc: Research support isp Octavio Ramilo, MD, Merck: Advisor/Consultant|Merck: Grant/Research Support|Merck: Honoraria|Moderna: Advisor/Consultant|Pfizer: Advisor/Consultant|Pfizer: Honoraria|Sanofi: Advisor/Consultant Asuncion Mejias, MD, PhD, MsCS, Enanta: Advisor/Consultant|Merck: Grant/Research Support|Moderna: Advisor/Consultant|Pfizer: Advisor/Consultant|Sanofi-Pasteur: Advisor/Consultant

